# Editorial: Exploring pigmentary disorders: pathogenesis to treatment

**DOI:** 10.3389/fmed.2026.1819040

**Published:** 2026-03-16

**Authors:** Vishal Thakur, Vignesh Narayan R

**Affiliations:** 1 Department of Dermatology and Venereology, All India Institute of Medical Sciences, Bathinda, India; 2 Department of Dermatology, Venereology and Leprology, St. Theresa Hospital, Bangalore, India

**Keywords:** autoimmunity, depigmentaion, melanoma, pigmentary disorder, vitiligo

Pigmentary dermatoses, encompassing disorders of hyperpigmentation, hypopigmentation, and depigmentation, represent a critical area of dermatological research due to their profound visible, systemic, and psychological impacts on patients. Although often perceived as primarily cosmetic, these disorders frequently reflect complex interactions between immunologic, genetic, metabolic, and environmental factors. Accordingly, advancing our understanding of pigmentary disease requires not only mechanistic insight but also translational strategies that bridge laboratory discovery with clinical implementation. This Research topic was established to decipher the complex pathogenic mechanisms underlying these conditions and to evaluate innovative therapeutic and diagnostic strategies. The contributing articles bridge the gap between bench research and clinical implementation, offering novel insights into autoimmune depigmentation and the early detection of pigmentary malignancies.

In the realm of autoimmune depigmentation, vitiligo remains a paradigmatic model for studying organ-specific autoimmunity. The contribution by Xie et al., *The fibroblast-driven melanoma/Treg vitiligo mouse model is effectively suppressed by IFN*γ *blocking antibody* highlights the critical orchestrating role of dermal fibroblasts in recruiting CD8+ cytotoxic T-cells. Utilizing a melanoma/Treg vitiligo mouse model, the authors established that the systemic administration of IFN-γ monoclonal antibodies significantly suppresses disease progression. The study positions this specific murine model as a robust, multi-antigen-specific platform for advancing the development of localized, fibroblast-targeted bispecific antibodies, potentially circumventing the infection risks associated with broad immunosuppressive agents.

Further exploring vitiligo pathogenesis, *Local sympathetic nerve depletion does not alter vitiligo progression in a mouse model* by Hu et al. rigorously investigates the longstanding clinical hypothesis connecting sympathetic nervous system hyperactivity to vitiligo onset. By utilizing 6-hydroxydopamine (6-OHDA) to induce chemical sympathectomy prior to vitiligo induction, the researchers observed that epidermal melanocyte loss and CD8+ T cell infiltration remained comparable to untreated control groups. This direct experimental evidence challenges prevailing theories, suggesting that previously observed sympathetic associations may be concomitant effects of the disease rather than causative drivers.

In response to these findings, Peng and Wang offer a critical methodological evaluation in their *Commentary: Local sympathetic nerve depletion does not alter vitiligo progression in a mouse model*. The authors note that while the study is a pioneering contribution, the melanoma-Treg model inherently depletes CD4+ T cells, thereby omitting crucial Th1 and Th17-mediated immune responses known to contribute directly to melanocyte destruction. To comprehensively evaluate the nervous system's involvement in vitiligo, the commentators recommend that future experimental designs incorporate explicit stress induction stimuli and utilize delayed observational time points to capture the dynamic changes following melanocyte stem cell depletion.

Beyond autoimmune conditions, this Research Topic also highlights the critical importance of diagnostic accuracy in malignant pigmentary disorders. Early detection of melanoma remains one of the most effective strategies for reducing morbidity and mortality. In *Melanoma toolkit for early detection for primary care clinicians: a 1-year follow-up on outcomes*, Diehl et al. evaluate a flexible, online continuing education program designed to enhance skin cancer screening capabilities among primary care providers (PCPs). The implementation of this multimedia curriculum—which allows users to tailor their learning via image recognition, videos, and clinical workflow solutions—resulted in significant improvements in PCPs' confidence and their diagnostic accuracy in distinguishing between malignant and benign lesions. This underscores the critical value of structured, adaptable educational interventions in expanding clinical screening capacities.

Collectively, the articles in this Research Topic advance our understanding of pigmentary dermatoses from the molecular signaling within localized fibroblasts to the optimization of front-line clinical diagnostics. They underscore the necessity of interdisciplinary collaboration, integrating immunology, neuroscience, oncology, and medical education. By continuing to explore these multifaceted conditions through a comprehensive, multidisciplinary approach, the dermatological community moves closer to delivering precise, evidence-based therapies that holistically improve patient outcomes and quality of life.

